# What is New about Neuroanaesthesia?

**Published:** 2009-08

**Authors:** G S Umamaheswara Rao

**Affiliations:** Professor, Department of Neuroanaesthesia, National Institute of Mental Health and Neurosciences, Bangalore 560 029

When Dr. John W. Michenfelder coined the term “Neuroanaesthesia” in his pioneering review published in 1969^1^, he would have least believed where the specialty has headed for in the past 40 years. From a service that provided anaesthesia to patients undergoing neurosurgery, it evolved to a subspecialty that combines the rapidly advancing basic and clinical neuroscience knowledge with the knowledge of anaesthesia to improve the outcomes of neurological patients. The speciality also has been able to contribute to the understanding of some of the basic phenomena in neurosciences. Seminal changes have occurred in the concepts in many clinical and research arenas as discussed below:

## Intraoperative Concerns during Craniotomy

Intraoperative concerns during craniotomy generally revolve around intracranial pressure (ICP) reduction and protection against inadvertent cerebral ischemia. Maintenance of optimal systemic physiology still remains the mainstay to achieve both the goals. Mannitol and hyperventilation have been the most commonly used tools for intraoperative ICP reduction. Hypertonic saline has emerged as a more physiological alternative to mannitol. But, the limited data available has not convincingly proved that it offers any advantage over mannitol for operative conditions of the brain during craniotomy^2^. With regards to hyperventilation, a recent study in patients undergoing surgery for supratentorial tumors has shown that moderate hyperventilation to a PaCO2 of 32-35 mmHg effects a significant change in subdural ICP and the surgeon's assessment of brain relaxation^3^. Whether such hyperventilation raises the same concerns of cerebral ischemia as in traumatic brain injury (TBI), remains to be evaluated.

Anaesthetics have been relied upon for intraoperative cerebral protection. Controversy continues regarding the superiority of one agent over others^4^. Inhalational agents have been claimed to offer more predictable protection than intravenous agents. Given the Experimental evidence that cerebral vascular occlusion caused under halothane anaesthesia produced smaller infarct volumes than under awake state raises the possibility that it is the anaesthetic state rather than the individual agent that offers protection. Apart from their effects on cerebral metabolism, anaesthetics seem to offer protection through other mechanisms such as ischemic preconditioning.

## Functional Neurosurgery and Awake Craniotomies

Awake craniotomy has become an option to preserve function during surgery in the proximity of eloquent areas of brain (speech area, motor area). Providing a calm and relaxed patient without the risk of airway obstruction is a challenge. Monitored anaesthesia care and asleep-awake-asleep techniques have been used with success.^5^,^6^

## Minimally Invasive Neurosurgery

Endoscopic neurosurgery with its advantages of minimal invasion and clear depiction of the structures is becoming more common^7^. Apart from surgical complications, of which vascular injury is the commonest, acute bradycardia and other arrhythmias including ventricular fibrillation have been documented^8^,^9^ during neuroendoscopy. Irrigation of the scope at high flows has been shown to increase the ICP to levels causing transient intracranial circulatory arrest and postoperative neurological complications^10^.

## Interventional Neuroradiology

Endovascular treatment with cyanoacrylate glues, Onyx liquid embolic system and detachable coils is rapidly becoming a routine choice for the management of cerebral aneurysms. Intra-arterial papaverine and nimodipine are being used in the treatment of cerebral vasospasm. General anaesthesia is preferred for these procedures. Laryngeal mask airway has been used as an alternative to endotracheal tube. Simple sedation, though used as an alternative to general anaesthesia, may pose the problems of airway obstruction and undesirable movement of the patient at crucial steps during the procedure^11^. Anaesthesiologists providing care during endovascular procedure may also have to deal with the haemorrhagic and ischemic cerebral complications that might occur during the procedure.

## Traumatic Brain injury

Traditionally, all efforts have been focused on optimising ICP in TBI. While there is some soundness in this approach, the need to look beyond ICP has been appreciated recently. Cerebral perfusion pressure (CPP)-based management is a step in this direction. After a great deal of debate on the appropriate level of CPP^12^, it is recognized that “one size does not fit all,” and CPP should be individually optimized to maximize the cerebral oxygenation and avoid anaerobic metabolism^13^. This calls for direct cerebral oxygen tension and cerebral metabolite monitoring. Technology required for this purpose is at present successful at experimental level and may soon be available freely for routine clinical use.

## Cerebral Ischemia

From cumbersome technologies suited only for experimental conditions, practical methods for bedside assessment of cerebral blood flow have been developed. With all its limitations, transcranial Doppler (TCD) remains an acceptable tool which is useful for repeated measurements. Its value has been established in subarachnoid haemorrhage, and TBI. Monitoring jugular venous oxygen saturation has provided insights into practical methods of optimizing global cerebral oxygenation, though it fails to provide regional information. Positron emission tomography (PET) and single photon emission computed tomography (SPECT) promise to provide deeper insights into ischemic mechanisms in future.

On a therapeutic front, management of cerebral ischemia still remains far from satisfactory. Once the ischemic is established, very few interventions have been proven to be of unequivocal benefit. Important among them is the emergency thrombolyis with rTPA in stroke^14^. Of some limited value are brain tissue O2 (PbtO2) guided management in TBI^15^ and hypertensive therapy in cerebral vasospasm^16^. Therapies based on single specific molecular mechanisms in the ischemic cascade have been uniformly unsuccessful.

## Benefits and Controversies beyond Neurosurgery

*Depth of Anaesthesia:* Knowledge of cerebral electrophysiology has prompted the development of monitors of depth of anaesthesia such as bispectral index (BIS)^17^, and spectral entropy^18^. Though questions have been raised on the consistency of their performance, and a recent study found no difference between BIS and end tidal anaesthetic agent concentration monitoring in preventing awareness during anaesthesia^19^, for the moment, they have brought in some measure of objectivity in the quantification of the depth of hypnosis.

*Mechanisms of Anaesthetic Action:* Functional studies through magnetic resonance imaging (MRI) and regional CBF/metabolism studies through PET open new vistas in understanding the mechanisms of anaesthetic action^20^

*Anaesthetics, Neurogenesis and Neurological Dysfunction:* *A*naesthetics have a potential to cause neurological dysfunction, which may take the form of postoperative neurocognitive dysfunction in the elderly, learning disabilities of the neonates exposed to anaesthetics in utero or early after birth, and precipitation or exacerbation of Alzheimer's in susceptible individuals^21^. Majority of this information comes from rodent experiments, the clinical relevance of which remains largely unexplored. There are a few clinical publications which are either retrospective analyses or case-control studies. The drugs that have been predominantly implicated in the causation of neurological dysfunction are bezodiazepines and ketamine, though evidence exists with other agents also including barbiturates, propofol, halothane, isoflurane, and sevoflurane.

The mechanism of anaesthetic-induced neurotoxicity remains unexplained. During normal CNS development, neurons are produced in excess and as much as 50%–70% of these neurons and progenitor cells undergo apoptosis^22^. Disruption of this physiological cell death mechanism seems to lead to intrauterine malformation of the brain and premature death of the embryo^23^. General anaesthetics seem to inhibit the synaptic transmission mediated through gamma aminobutyrate (GABA) and/or *N*-methyld-aspartate (NMDA) receptors. Because GABA-and NMDA-mediated neuronal activity is essential for mammalian brain development, exposure to general anaesthetics could potentially interfere with normal brain maturation^24^‐^26^.

In conclusion, neumanaesthesia has evolved from a clinical service catering to the needs of neurological patients to a scientific discipline that is also exploring the basics of nervous system with benefits extending beyond anaesthesia to neurosciences in general. Paradoxically, the goal of generating large clinical evidence-base to formulate practice guidelines remains unfulfilled. Given the voluminous clinical material available in India, it should be possible for us to undertake this task in our country!
